# Serotonin transporter gene, childhood emotional abuse and cognitive vulnerability to depression

**DOI:** 10.1111/j.1601-183X.2010.00593.x

**Published:** 2010-08

**Authors:** N Antypa, A J W Van der Does

**Affiliations:** † Institute of Psychology, Leiden UniversityLeiden, The Netherlands; ‡ Department of Psychiatry, Leiden University Medical CentreLeiden, The Netherlands

**Keywords:** childhood emotional abuse, cognitive reactivity, depression, neuroticism, serotonin transporter gene

## Abstract

Meta-analyses evaluating the association between the serotonin transporter polymorphism (5-HTTLPR) with neuroticism and depression diagnosis as phenotypes have been inconclusive. We examined a gene–environment interaction on a cognitive vulnerability marker of depression, cognitive reactivity (CR) to sad mood. A total of 250 university students of European ancestry were genotyped for the 5-HTTLPR, including SNP rs25531, a polymorphism of the long allele. Association analysis was performed for neuroticism, CR and depression diagnosis (using a self-report measure). As an environmental pathogen, self-reported history of childhood emotional abuse was measured because of its strong relationship with depression. Participants with the homozygous low expressing genotype had high CR if they had experienced childhood emotional maltreatment but low CR if they did not have such experience. This interaction was strongest on the Rumination subscale of the CR measure. The interaction was not significant with neuroticism or depression diagnosis as outcome measures. Our results show that 5-HTTLPR is related to cognitive vulnerability to depression. Our findings provide evidence for a differential susceptibility genotype rather than a vulnerability genotype, possibly because of the relatively low levels of abuse in our sample. The selection of phenotype and environmental contributor is pivotal in investigating gene–environment interactions in psychiatric disorders.

One of the most studied polymorphisms in psychiatry is in the promoter region of the serotonin transporter gene (5-HTTLPR). A recent meta-analysis concluded that there is no association between this polymorphism and depression, and also no interaction effect with stressful life events (Risch *et al.* 2009). This meta-analysis has major methodological limitations (Rutter *et al.* 2009), and an updated, comprehensive review provides further evidence of the association (Uher & McGuffin 2010). The inconsistencies in findings may be as a result of the heterogeneity of both the phenotype (depression) and the environment (life events) (Lotrich & Lenze 2009). Some of the largest studies may have been atypical because these did not show the well-documented association between stressful life events and depression (Gillespie *et al.* 2005; Surtees *et al.* 2006). Furthermore, depression as defined by DSM-IV is a heterogeneous concept and therefore may not be the most promising outcome for genetic research (Jacobs *et al.* 2006). Continued investigation of the relationship between 5-HTTLPR and depression is also warranted because of its association with emotion regulation and social cognition (Canli *et al.* 2007) and with amygdala activity (Munafo *et al.* 2008).

Studies on the association of 5-HTTLPR with the personality trait of neuroticism have also produced inconsistent results (Munafo *et al.* 2009). Most studies have failed to take the moderating effects of childhood trauma or life events into consideration. Moreover, longitudinal research has shown that neuroticism scores may simply reflect the average level of distress over a protracted period (Ormel *et al.* 2004), and thus may lack specificity for depression.

Cognitive reactivity (CR) to sad mood may be a more promising vulnerability measure of depression than neuroticism, and does not have the limitations of a categorical diagnosis of depression. The concept of CR is based on the differential activation theory, which states that conditioned associations that are formed between depressed mood states and dysfunctional cognitions remain intact during periods of remission (Teasdale 1988). These cognitions may then be re-activated by relatively small changes in mood, and may even be present in vulnerable samples before the onset of a first episode of depression. The extent to which maladaptive cognitions are triggered by (non-pathological) low mood is referred to as CR (Scher *et al.* 2005). Experimental studies using sad mood inductions have shown that formerly depressed patients have higher CR than never-depressed individuals (Segal *et al.* 1999). High CR increases the risk of depressive relapse, independently from prior treatment (Segal *et al.* 2006). A self-report measure of CR [the Leiden Index of Depression Sensitivity-Revised (LEIDS-R)] contains several subscales that cover several dimensions of maladaptive cognitions that characterize the vulnerable depressive mind (see *Methods*). It reliably distinguishes between previously depressed and never-depressed groups and also correlates highly with CR as measured with a mood induction procedure (Van der Does 2002). The LEIDS-R, but not neuroticism, predicts response to serotonin depletion (Booij & Van der Does 2007), which is a potential endophenotype of depression (Hasler *et al.* 2004). LEIDS-R scores were also found to have a unique contribution to the prediction of depression, over and above trait level of depressive rumination (Moulds *et al.* 2008). Furthermore, LEIDS-R scores mediate the relationship between neuroticism and depressive symptomatology in both never-depressed and previously depressed groups (Barnhofer & Chittka 2010).

We investigated whether the association of 5-HTTLPR with CR would be stronger than with neuroticism or with depression diagnosis. We hypothesized that this relationship would be moderated by childhood abuse, in particular childhood *emotional* abuse (CEA). Rose and Abramson (1992) suggested that emotional abuse is more likely to contribute to a depressogenic cognitive style because the child receives negative cognitions by the abuser in a direct manner: ‘you are worthless'. In this way, the child tends to adopt a general negative attributional style, which contributes to the development of depression. For other types of maltreatment, the child has to make his or her own attributions, which may allow more room for less global and more external attributions. Empirical studies support this theory and show that CEA is differentially associated with depression compared with other types of maltreatment (Chapman *et al.* 2004; Gibb *et al.* 2001, 2003, 2007; Hovens *et al.* in press). Prospective studies also indicate that CEA predicts symptoms or diagnosis of depression (Gibb *et al.* 2001; Hankin 2005; Liu *et al.* 2009). Furthermore, there is evidence that the relationship between CEA and depressive symptoms is mediated by cognitive factors (Gibb *et al.* 2001; Hankin 2005; Raes & Hermans 2008; Wright *et al.* 2009).

## Methods

### Participants and procedure

We tested the Gene × Environment (G × E) hypothesis in 250 university students of European ancestry. The research was approved by the Ethics Committee of the Leiden University Medical Center in the Netherlands and all participants gave written informed consent before participating in the study.

DNA was obtained using the Oragene Self-Collection Kit—DISC format (DNA Genotek Inc, Ottawa, ON, Canada); 200 µl of saliva was collected in lysis buffer (100 mm NaCl, 10 mm EDTA, 10 mm Tris pH 8, 0.1 mg/ml proteinase K and 0.5% w/v SDS) until further processing.

#### DNA isolation

Genomic DNA was isolated from the samples using the Chemagic kit on a Chemagen Module I workstation (Chemagen Biopolymer-Technologie AG, Baesweiler, Germany). DNA concentrations were quantified by OD260 measurement and by agarose gel electrophoresis. The average yield was approximately 4 µg of genomic DNA per sample.

#### Polymerase chain reaction amplification

The region of interest from the serotonin transporter (5-HTT) gene was amplified by triplex polymerase chain reaction (PCR) using the following primers: a FAM-labelled primer HTTLPR-FWFAM 5′-TCCTCCGCTTTGGCGCCTCTTCC-3′, and a reverse primer HTTLPR-RV 5′-TGGGGGTTGCAGGGGAGATCCTG-3′. Typical PCR reactions contained between 10 and 100 ng of genomic DNA template, and 10 pmol of forward and reverse primer. PCR was carried out in the presence of 5% DMSO with 0.5 U of BioThermAB polymerase (GeneCraft, Munster, Germany) in a total volume of 30 µl using the following cycling conditions: initial denaturation step of 5 min at 95°C, followed by 40 cycles of 30 seconds at 96°C, 30 seconds at 61°C, 60 seconds 72°C and a final extension step of 10 min at 72°C. After PCR, 5 µl of the sample was subjected to restriction digestion with the enzyme HpaII in a total volume of 20 µl. Restriction was incubated for 3 h at 37°C.

#### Analysis of PCR products

One microlitre of PCR product before and after restriction digestion was mixed with LIZ-500 size standard and formamide and run in two separate lanes on an AB 3100 genetic analyser set up for genotyping with 50 cm capillaries. Results were analysed using Genescan software version 3.7 (Applied Biosystems, Carlsbad, CA, USA), and alleles were scored visually according to the following scheme: Uncut: S, 469 bp; L, 512 bp. Cut: Sg, 402 + 67 bp; Lg, 402 + 110 bp.

Genotype analysis failed for two participants, yielding 248 samples for association analysis. Genotype frequencies were as follows: SS, 16.9%; SLg, 5.2%; LgLg, 0.8%; LaLg, 8.9%; SL, 37.1%; LaLa, 31.1%. Participants were divided on the basis of the triallelic classification (Lg alleles were collapsed with ‘s’ variants according to evidence of similar functionality) into three genotype groups: S′S′ (*n* = 57); L′S′ (*n* = 114); L′L′ (*n* = 77). Genotype frequencies were consistent with Hardy–Weinberg Equilibrium *χ*^2^(1) = 1.55, *P* = 0.21.

### Measures

CR was measured using the LEIDS-R (Van der Does 2002, 2005; Williams *et al.* 2008). Participants are asked to indicate whether and how their thinking patterns change when they experience mild dysphoria, by scoring each item on a 5-point Likert scale ranging from 0 ‘not at all’ to 4 ‘very strongly’ applicable. The LEIDS-R has 34 items and covers six subscales: *Hopelessness/Suicidality* (‘When I feel down, I more often feel hopeless about everything’); *Acceptance/ Coping* (‘When I am sad, I feel more like myself’); *Aggression* (‘When I feel down, I lose my temper more easily’); *Control/Perfectionism* (‘When in a sad mood, I become more bothered by perfectionism’); *Risk Aversion* (‘When I feel down, I take fewer risks'); *Rumination* (‘When I feel sad, I spend more time thinking about the possible causes of my moods'). Internal consistency (Cronbach's *α*) for the LEIDS-R total was 0.89, and ranged between 0.62 and 0.83 for the subscales.

Neuroticism was assessed with the Dutch/Flemish authorized translation of the 60-item NEO PI-R (Hoekstra *et al.* 2007); Cronbach's *α* was 0.88. The presence of current and past depression was assessed with the Major Depression Questionnaire. The measure covers all DSM-IV diagnostic criteria for current and past major depression. Consistency of this questionnaire with diagnoses based on structured clinical interview for DSM disorders (SCID) has been examined in a sample of 39 individuals: *Sensitivity* = 100%; *Specificity* = 75%; *Positive Predictive Value* = 79%; *Negative Predictive Value* = 100%; overall *κ* = 0.75) (Williams *et al.* 2008).

CEA was measured with the Childhood Trauma Questionnaire (28-item version), which is a screening measure for maltreatment histories in both clinical and non-referred groups (Bernstein & Fink 1998). The emotional abuse subscale refers to verbal assaults on a child's sense of worth or well-being, or any humiliating and demeaning behaviour directed towards a child by an older person (Bernstein & Fink 1998). Total CEA has a range of 5 (no abuse) to 25, and internal consistency in this sample was *α* = 0.80. An example item from the CEA scale is ‘People in my family said hurtful or insulting things to me’.

Current symptoms of anxiety and depression were used as a covariate. An authorized Dutch translation of the Hospital Anxiety and Depression Scale (HADS), a 14-item self-report screening scale for anxiety and depression, was used (Spinhoven *et al.* 1997). Internal consistency for the total score was *α* = 0.87.

### Statistical analysis

SPSS 16.0 was used for data analysis. Data were screened for accuracy and normal distribution assumptions. Square root transformations corrected outlying data (*z >* 3) and heterogeneity of variance on the LEIDS-R total and subscales. Figures report untransformed values. We used analysis of variance (ANOVA) to detect main effects and interactions on continuous outcomes—Univariate ANOVA was used for the LEIDS-R and neuroticism total scores and a separate multivariate ANOVA for the LEIDS-R subscales, because of high correlations with the total score. Partial eta squared (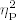) is reported as an estimate of effect size. We used logistic regression for depression diagnosis (none vs. lifetime) as an outcome. Independent variables were the 5-HTTLPR genotype (coded 1 for S′S′, coded 2 for S′L′, coded 3 for L′L′) and the CEA (median split: above score 6 coded as 1, below or equal to score 6 coded as 0). Additional analyses were conducted with current depression and anxiety (HADS total score) as covariates, as current levels of symptomatology can affect CR (Williams *et al.* 2008). We re-ran the analyses with physical abuse for purposes of adversity specificity, and also with the biallelic genotype classification.

## Results

Table 1 displays the sample characteristics. Among genotype groups, no differences were found with respect to age (*F*_2,245_ = 1.2, *P* = 0.30) and gender distribution [*χ*^2^(2) = 1.11, *P* = 0.58]. No between-group differences were found on depression diagnoses [*χ*^2^(4) = 1.68, *P* = 0.80]. There were also no differences between genotypes in current levels of anxiety (HADS anxiety subscale) (*P* = 0.94), or depression (HADS depression subscale) (*P* = 0.61), or total symptomatology (HADS total) (*F*_2,245_ = 0.20, *P* = 0.82). Genotype groups also did not differ on CEA scores (*F*_2,245_ = 2.44, *P* = 0.09) (*post hoc* tests also non-significant).

**Table 1:** Participant characteristics by 5-HTTLPR genotype (*N* = 248)

Genotype	S′S′ (*N* = 57)	S′L′ (*N* = 114)	L′L′ (*N* = 77)
Age (mean ± SD)	23.3 ± 6.1	22.5 ± 4.2	22.0 ± 4.5
Females	77.2%	72.8%	79.2%
Depression diagnosis (%)			
No lifetime MDD	56.1	61.4	63.6
Past MDD	38.6	31.6	28.6
Current MDD	5.3	7.0	7.8
Current symptoms (mean ± SD)			
HADS total	9.4 ± 5.8	8.8 ± 5.9	9.1 ± 5.3
HADS depression	2.9 ± 3.1	2.5 ± 2.8	2.8 ± 3.0
HADS anxiety	6.4 ± 3.3	6.3 ± 3.7	6.2 ± 3.0
Childhood emotional abuse (mean ± SD)	8.2 ± 3.1	7.5 ± 3.4	7.0 ± 2.9

HADS, Hospital Anxiety Depression Scale; MDD, major depressive disorder.

**Figure 1:**
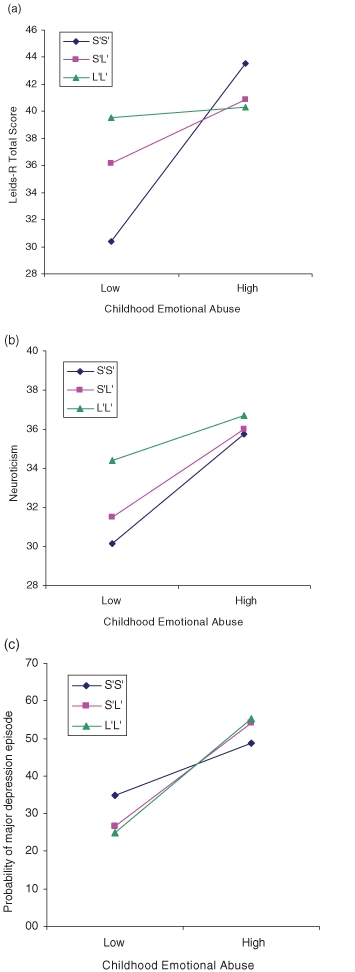
**Gene–environment (G × E) interactions on the three depression-related outcomes.** (a) G × E interaction on cognitive reactivity (LEIDS-R total score), *P* = 0.04; (b) G × E interaction on neuroticism, *P* = 0.54; (c) G × E interaction on depression diagnosis (proportion), *P* = 0.59.

ANOVA yielded a significant interaction between genotype and emotional abuse on CR (*F*_2,242_ = 3.21, *P* = 0.04) (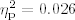). The S′S′ genotype scored the lowest in a low abuse environment (Fig. 1a) (*F*_2,129_ = 3.4; *P* = 0.035). *Post hoc* Tukey test showed that when CEA was low, the S′S′ genotype had significantly lower scores than the L′L′ genotype (*P* = 0.03). When CEA was high, differences between genotype groups failed to reach significance. Furthermore, CEA was associated with higher CR scores (*F*_1,242_ = 6.56, *P* = 0.01) (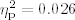), but there was no direct association between genotype and CR (*F*_2,242_ = 0.492, *P* = 0.61) (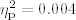). The same interaction was also significant for the Rumination subscale of the LEIDS-R (*F*_2,242_ = 4.143, *P* = 0.017) (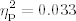). The S′S′ genotype again showed the highest scores under high childhood emotional maltreatment, but the lowest scores under low maltreatment. *Post hoc* tests for each maltreatment group again showed that when CEA was low, the S′S′ genotype had significantly lower scores than the L′L′ (*P* = 0.02); no other significant differences were found. A similar interaction was marginally significant for the Risk Aversion subscale (*F*_2,242_ = 2.920, *P* = 0.056) (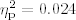). After entering current symptoms of anxiety and depression as a covariate, the interactions became stronger and were all significant (*P <* 0.05). We examined correlations between CEA and CR for each genotype separately. Within the S′S′ genotype, we found significant correlations between CEA and the LEIDS-R total score (*r* = 0.28, *P* = 0.04), and with the Rumination subscale (*r* = 0.30, *P* = 0.03). Within the S′L′ genotype, we found a significant correlation only with the total score (*r* = 0.20, *P* = 0.03). There were no significant correlations within the L′L′ genotype group.

We found no significant G × E interaction on neuroticism (*F*_2,242_ = 0.62, *P* = 0.54) (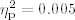) (Fig. 1b) with or without covariates. There was a main effect of emotional abuse (*F*_1,242_ = 12.39, *P* = 0.001) (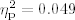), but no main effect of genotype (*F*_2,242_ = 1,62, *P* = 0.20) (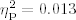). Using probability of depression diagnosis (current and/or past) as outcome, we also found a main effect of emotional abuse (*P* = 0.009) but no main effect of genotype and no interaction (*P >* 0.58) (Nagelkerke *R*^2^ = 0.094) (Fig. 1c). To determine specificity of adversity, main analyses were run with physical abuse, rather than emotional abuse, as the environmental contributor. All G × E interactions were non-significant for all outcome measures.

### Biallelic analyses

Genotype groups were re-classified as SS vs. SL vs. LL, not taking into account the rs25531 SNP. Using the *biallelic* classification, observations were consistent with HWE: *χ*^2^(1) = 3.29, *P* = 0.07. After controlling for current symptoms of depression and anxiety (HADS total), the same results were found as with the triallelic classification [G × E interaction: RUM: (*F*_2,241_ = 3.95, *P* = 0.02) LEIDS-R total: (*F*_2,241_ = 4,03, *P* = 0.02)]. G × E interactions fell short of statistical significance for the uncorrected analyses (RUM: *P* = .13; LEIDS-R: *P* = 0.33). However, correlations between emotional abuse and the LEIDS-R outcomes for each genotype separately remained significant, consistent with the results for the triallelic classification. G × E interactions were non-significant for the other outcomes.

## Discussion

Our results show that 5-HTTLPR moderates the effect of CEA on CR, a cognitive vulnerability factor of depression. Participants with the SS (including Lg) genotype with a low emotional abuse history had significantly lower CR scores than the other genotype groups. They also had (non-significantly) higher CR than other genotypes when emotional abuse in childhood was above threshold. After controlling for current levels of depression and anxiety symptoms, the G × E interactions become stronger and were replicated in the analysis using the biallelic classification. The LEIDS-R is designed to capture negative cognitive patterns of reactivity, which are *habitual* in nature. In vulnerable individuals, LEIDS-R scores remain high when symptoms are low, but the scores do tend to get higher with increasing levels of depression. In other words, the scores are partly mood-dependent, which makes it important to rule out variance explained by current state. The gene–environment interaction on the Rumination subscale of the LEIDS-R is noteworthy, as rumination is a robust vulnerability factor for (and characteristic of) depression (Nolen-Hoeksema 2000). The same subscale was also found to mediate the relationship between neuroticism and depressive symptoms in never-depressed individuals (Barnhofer & Chittka 2010). Furthermore, a similar crossover interaction of the 5-HTTLPR and life stress has been previously found with another measure of rumination (Canli *et al.* 2006).

This pattern of a crossover interaction on CR supports the ‘differential susceptibility’ theory that states that individuals of a certain genetic make-up are not merely vulnerable to an adverse outcome but rather ‘susceptible’: they are more likely to suffer from an adverse environment but also benefit more from a supportive one (Belsky *et al.* 2009). The same differential susceptibility pattern of the SS genotype across childhood experiences may be observed in a number of previous reports (Eley *et al.* 2004; Kaufman *et al.* 2004; Taylor *et al.* 2006; Wilhelm *et al.* 2006). In our study, the positive effect of the SS genotype in low childhood maltreatment environments seems to be higher than the adverse effect of the same genotype when having experienced high maltreatment. Similar magnitude of differential effects has been found in previous research (Eley *et al.* 2004; re-evaluated in Belsky *et al.* 2009). A possible explanation for this pattern is that in our sample CEA was within the lower quartiles, allowing less variability for detecting higher vulnerability to adversity.

Limitations of the present study include the retrospective assessment of childhood abuse using a self-report measure, which may have led to a biased reporting. However, the study design may actually be rather conservative for detecting G × E interactions because emotionally abused individuals are underrepresented in our young student sample (i.e. restricted range on E variable), and abused individuals may have failed to recollect their maltreatment (i.e. mis-specification of E variable). Another limitation is that we were unable to use the established cut-off scores for dichotomizing CEA because of the relatively low mean scores in our sample. Future studies examining samples with higher exposure to maltreatment may detect genotype vulnerability effects of greater magnitude, especially in the *high* emotional abuse quartiles. Moreover, our assessment of depression diagnosis by self-report is questionable. Although validity data for all self-report measures are available, these results should be interpreted with caution.

Our sample was comprised of young adults, which is consistent with previous studies that successfully detected a gene–environment interaction on depression outcomes (Caspi *et al.* 2003; Eley *et al.* 2004; Taylor *et al.* 2006) vs. those who did not (Gillespie *et al.* 2005; Power *et al.* in press; Surtees *et al.* 2006). The advantage of using CR as an outcome measure—as opposed to depression history—is that CR can also be assessed in people who are vulnerable to depression but have not yet experienced an episode of depression. Many genetic associations to brain-based endophenotypes are also observed in healthy individuals (Meyer-Lindenberg & Weinberger 2006). Finally, it should be noted that our sample size is small for the detection of gene–environment interactions. Our sample is also not representative of the general population; hence, the generalizability of our findings remains to be investigated. If replicated, our results show that the strength of the association between 5-HTTLPR and depression partly depends on the selection of the environmental pathogen and the marker of depression vulnerability.
